# A study on the consequence of swift heavy ion irradiation of Zn–silica nanocomposite thin films: electronic sputtering

**DOI:** 10.3762/bjnano.5.179

**Published:** 2014-10-01

**Authors:** Compesh Pannu, Udai B Singh, Dinesh C Agarwal, Saif A Khan, Sunil Ojha, Ramesh Chandra, Hiro Amekura, Debdulal Kabiraj, Devesh K Avasthi

**Affiliations:** 1 Inter-University Accelerator Centre, Aruna Asaf Ali Marg, New Delhi 110067, India; 2 Institute Instrumentation Centre, Indian Institute of Technology, Roorkee 247667, India; 3 National Institute for Materials Science (NIMS), Tsukuba, Ibaraki 305-0003, Japan

**Keywords:** ion irradiation, nanocomposites, pressure spike, Rutherford backscattering spectrometry, transmission electron microscopy

## Abstract

Zn–silica nanocomposite thin films with varying Zn metal content, deposited by atom beam sputtering technique were subjected to 100 MeV Ag ion irradiation. Rutherford backscattering spectrometry reveals the loss of Zn with irradiation, which is observed to be greater from thin films with lower Zn content. The sputtered species collected on carbon-coated transmission electron microscopy (TEM) grids consist of Zn nanoparticles of sizes comparable to those present in the nanocomposite thin film. The process of size-dependent electronic sputtering of Zn is explained on the basis of an inelastic thermal spike model. The possibility of direct cluster emission is explained by pressure spike built inside the track**,** initiated by a temperature spike.

## Introduction

Metal nanoparticles are currently receiving broad scientific and technological interest due to their unusual physical properties which are different from the bulk materials. Properties such as surface plasmon resonance [1], fast optical response [2], and superparamagnetism [3], strongly depend on shape, size, size distribution and the surrounding environment of the metal nanoparticles [4]. Thus, the properties of nanocomposites can be controlled by the variation of these parameters. Swift heavy ion (SHI) irradiation is an effective tool to engineer the properties of the nanocomposite thin films. SHI produces electronic excitations in a narrow cylindrical volume around its path, leading to a change in size and shape of metal nanoparticles embedded in a matrix [5–8]. The group at IUAC [5] showed that growth as well as a reduction in size of nanoparticles occur upon SHI irradiation, depending on the size of nanoparticles and the interparticle separation. Awazu et al. [9] reported an upper limit and lower limit for the elongation of nanoparticles. It is observed that ion irradiation raises the track temperature above the melting temperature of gold and silica for nanoparticles with small radii, but in the case of larger nanoparticles gold does not melt.

In SHI irradiation of nanocomposite thin films, along with change in size and shape of nanoclusters, the phenomenon of sputtering also occurs. It has potential applications in astrophysics, space devices and mass spectrometry of large molecules [10]. Sputtering occurs due to the energy transfer to the atoms in the target by incident ions through nuclear collision cascades (nuclear sputtering) produced in the target as well as electronic interactions (electronic sputtering). The former and the latter, respectively, work mainly at low and high energy regimes. Nuclear sputtering (in the kiloelectronvolt energy regime) was studied widely in many different materials and is well explained by Sigmund’s theory [11]. According to this theory, sputtering of a material due to ion irradiation results from a cascade of atomic collisions. At high energy or in the electronic energy loss regime, a large increase in sputtering yield has been reported by several groups [12–16]. Sigmund’s theory fails to explain the reported large increase in sputtering yield [17]. Several mechanisms were proposed to describe the large sputtering yield, such as shock wave model [18], thermodynamical equilibrium model [19], ion explosion, thermal spike [20] and even a combination of ion explosion and thermal spike [21]. In the case of metals, the electronic sputtering yield is not so high but definitely larger than predicted by Sigmund’s theory [13]. Mieskes et al. [16] studied the sputtering of insulator and metal targets in the electronic stopping regime and observed that metals exhibit sputtering rates that are two to three orders of magnitude lower than that of insulators. The low sputtering rate of metals is due to the high mobility of the conduction electrons, which quickly smear out the deposited energy. The large sputtering yield in insulators is well beyond the collision cascade theory, which assigns sputtering mainly to electronic excitations. But in case of metals, experimentally measured sputtering yields indicate a synergetic effect of electronic excitations and nuclear collision cascades.

The emission of clusters raised great scientific interest in the field of ion beam interaction with matter in order to understand the fundamental processes involved during sputtering. In particular, much interest has been devoted to determine the sputtering yield in different materials in dependence on various ions and energies. But there are not many reports which simultaneously studied the emission of clusters and determined the sputtering yield. Honig et al. [22] first reported the emission of clusters of atoms during sputtering. It is well known that sputtered particles contain clusters of several atoms as well as single atomic species [23]. The direct knock-out of clusters and sputtering in the form of atomic species have been suggested by several authors. Rehn et al. [24] reported the ejection of stable clusters during ion irradiation and this observation of stable clusters was surprising because the binding energy of the clusters is smaller than the energy deposited by the ion in the material. A shock wave model [18] was proposed to explain the emission of larger clusters. Several studies have been carried out to understand the basic mechanism behind the emission of stable clusters [23–25], while any decisive mechanism is yet to be proposed.

The sputtering yield in bulk metals is reported to be only a few atoms/ions in the electronic energy loss regime [16]. However as we proceed from bulk to nanodimensional system, materials properties, such as melting point and surface to volume ratio, change drastically. Therefore, sputtering is expected to be appreciable and affected by several parameters such as substrate, grain size and thickness of film. Gupta and Avasthi [26] studied the electronically mediated sputtering of thin gold films and observed that electronic sputtering increases with a decrease in the thickness of the film. Singh et al. [27] reported that the electronic sputtering from Ag thin films is three orders of magnitude higher than the expected value for bulk Ag. Sputtering from thin films through SHI was studied widely [26–28], but very few reports are available on the sputtering from nanocomposite thin films. In case of nanocomposite thin films, Singh et al. [29] reported a dependence of the sputtering yield of Au from Au–silica nanocomposite on the size of Au nanoparticles.

To the best of our knowledge, no reports are available on sputtering of Zn nanoparticles embedded in silica matrix by using SHI. Zn nanoparticles have great potential for application in several fields. These can be used in cancer therapy, anticorrosive coating and antimicrobial coatings, in chemical reaction as catalyst, galvanization of iron and steel [30–31]. Bulk Zn has a melting point of 419.6 °C and the melting point of Zn nanoparticle varies from 250 to 420 °C depending on the size of nanoparticle [32–33]. The melting point of Zn is much lower than that of other metals like Au, Ag, Cu and Pt. The melting point is considered to play an important role in sputtering of nanoparticles by using SHI irradiation. The present work is a study of SHI-induced sputtering of Zn nanoparticles embedded in silica matrix and its dependence on the size of nanoparticles. Apart from electronic sputtering, the sputtered species collected on a catcher are also studied. In this report, an effort is made for understanding the interaction of the ion beam with nanodimensional material in light of the results obtained on the sputtering process and emission of clusters during ion irradiation.

## Experimental

**Figure 1:**
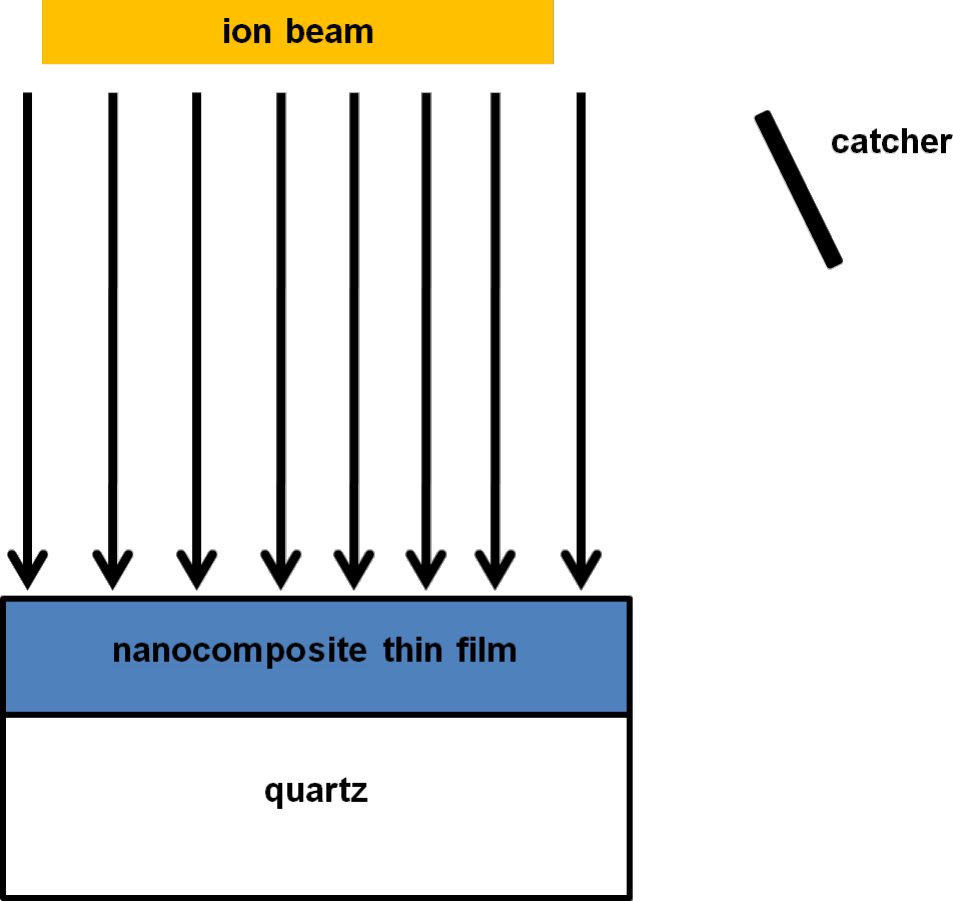
Experimental setup. The ion beam is incident perpendicularly to the nanocomposite thin film and catcher is placed at an angle of 60° from thin film surface.

Two sets of Zn–silica nanocomposite thin films were deposited on quartz and TEM grids by using atom beam sputtering [34–36]. One set of samples, referred to as set A, has 2 atomic % Zn in silica and the other set of films, referred to as set B, has 10 atomic % Zn in silica. The thicknesses of the nanocomposite thin films for samples A and samples B are about 280 nm and about 60 nm, respectively. One half of each sample of the two sets was irradiated and the other half was treated as pristine. The irradiation was performed with 100 MeV Ag^7+^ ions at room temperature by using the 15 UD Pelletron accelerator facility at Inter University Accelerator Centre, New Delhi. The ion flux during irradiation was 1.8 × 10^10^ ions/(cm^2^·s). The samples were irradiated with fluences of 1 × 10^13^ and 3 × 10^13^ ions/cm^2^ and the ions were incident perpendicularly to the thin films. The pressure in the chamber during irradiation was about 10^−7^ mbar. Simultaneously, the sputtered particles were collected on carbon-coated TEM grids, mounted 1.5 cm above the target at an angle of 60° with respect to the sample surface. The schematic of experimental setup is shown in Figure 1. Rutherford backscattering spectrometry was performed to determine the Zn content in the silica of pristine and irradiated samples by using the 1.7 MV tandem accelerator facility at IUAC, New Delhi with 2 MeV He^+^ ions. A silicon surface barrier detector was used at a backscattering angle of 170°. The sputtered particles collected on the catcher (TEM grids) were examined by TEM to determine the size distribution of the nanoparticles. TEM measurements were performed by using a 200 kV FEI TECHNAI 20 TEM at Institute Instrumentation Centre, Indian Institute of Technology, Roorkee.

## Results

**Figure 2:**
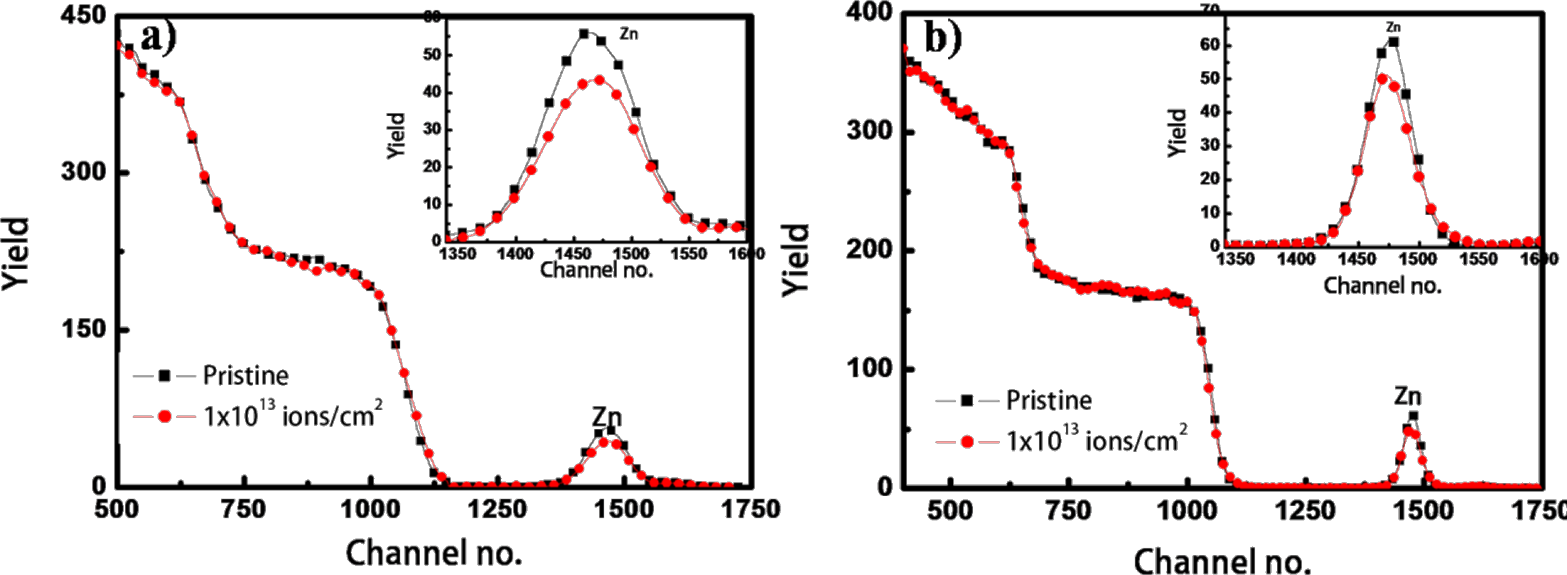
RBS spectra of Zn–silica nanocomposite thin film before and after irradiation, (a) 2 atomic % Zn in silica, (b) 10 atomic % Zn in silica. The inset in both figures shows the zoomed Zn peak for clarity.

RBS spectra of the pristine and the irradiated samples are shown in Figure 2a and Figure 2b, respectively. The integrated area under the Zn peak in RBS spectra is used to determine the Zn content. The Zn content in case of set A is found to be (2.65 ± 0.05) × 10^16^ atoms/cm^2^ for pristine and (2.32 ± 0.04) × 10^16^ atoms/cm^2^ for irradiated sample. Similarly, the Zn content in case of set B is found to be (1.82 ± 0.04) × 10^16^ atoms/cm^2^ for pristine and (1.55 ± 0.04) × 10^16^ atoms/cm^2^ for the irradiated sample. Thus, a reduction in the areal concentration of Zn is clearly observed after irradiation. The difference in the areal concentration is used to determine the sputtering yield of Zn atoms by using the relation

[1]
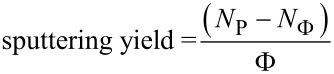


where Φ is irradiation fluence, *N*_Φ_ is the areal concentration of atoms after irradiation and *N*_P_ is the areal concentration of atoms in the pristine film. The sputtering yield of Zn is found to be 330 atoms/ion for set A and 270 atoms/ion for set B, whereas the sputtering from bulk Zn for 100 MeV Ag ions is 0.4 atoms/ion calculated through the simulation software TRIM (transport of ions in matter) [37]. In the present case sputtering yield is about three orders of magnitude higher than that encountered in the corresponding bulk metal in the nuclear energy loss regime.

**Figure 3:**
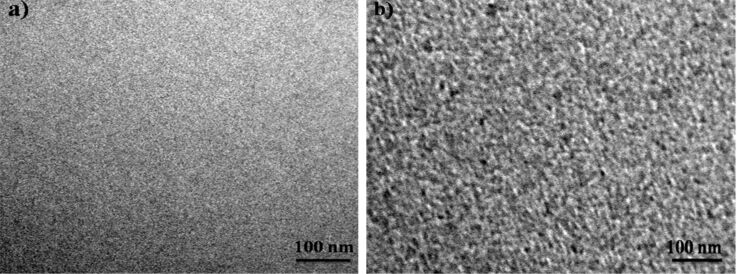
TEM micrographs of 2 atomic % Zn in silica, (a) pristine film, (b) irradiated at a fluence of 3 × 10^13^ ions/cm^2^.

**Figure 4:**
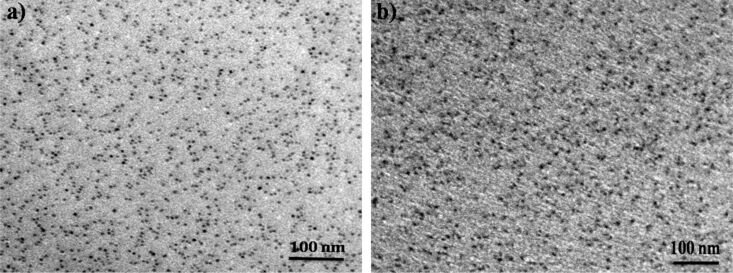
TEM micrographs of 10 atomic % Zn in silica, (a) pristine film, (b) irradiated at a fluence of 3 × 10^13^ ions/cm^2^.

TEM measurements were carried out on the pristine and irradiated nanocomposite films on carbon-coated TEM grids. The micrographs of sample set A before and after irradiation are shown in Figure 3a and Figure 3b, respectively. The pristine film is likely to have Zn in the atomic state itself or may have small Zn clusters and the formation of nanoparticles of a size around 5.5 nm takes place during irradiation. The nucleation and growth of metal nanoparticles in silica is well established and reported earlier [38]. TEM micrographs of pristine and irradiated films of sample set B are shown in Figure 4a and Figure 4b, respectively. The average size of nanoparticles in the pristine film is 6.7 nm and after irradiation it is around 7.7 nm, indicating slight change in size of nanoparticles during irradiation.

**Figure 5:**
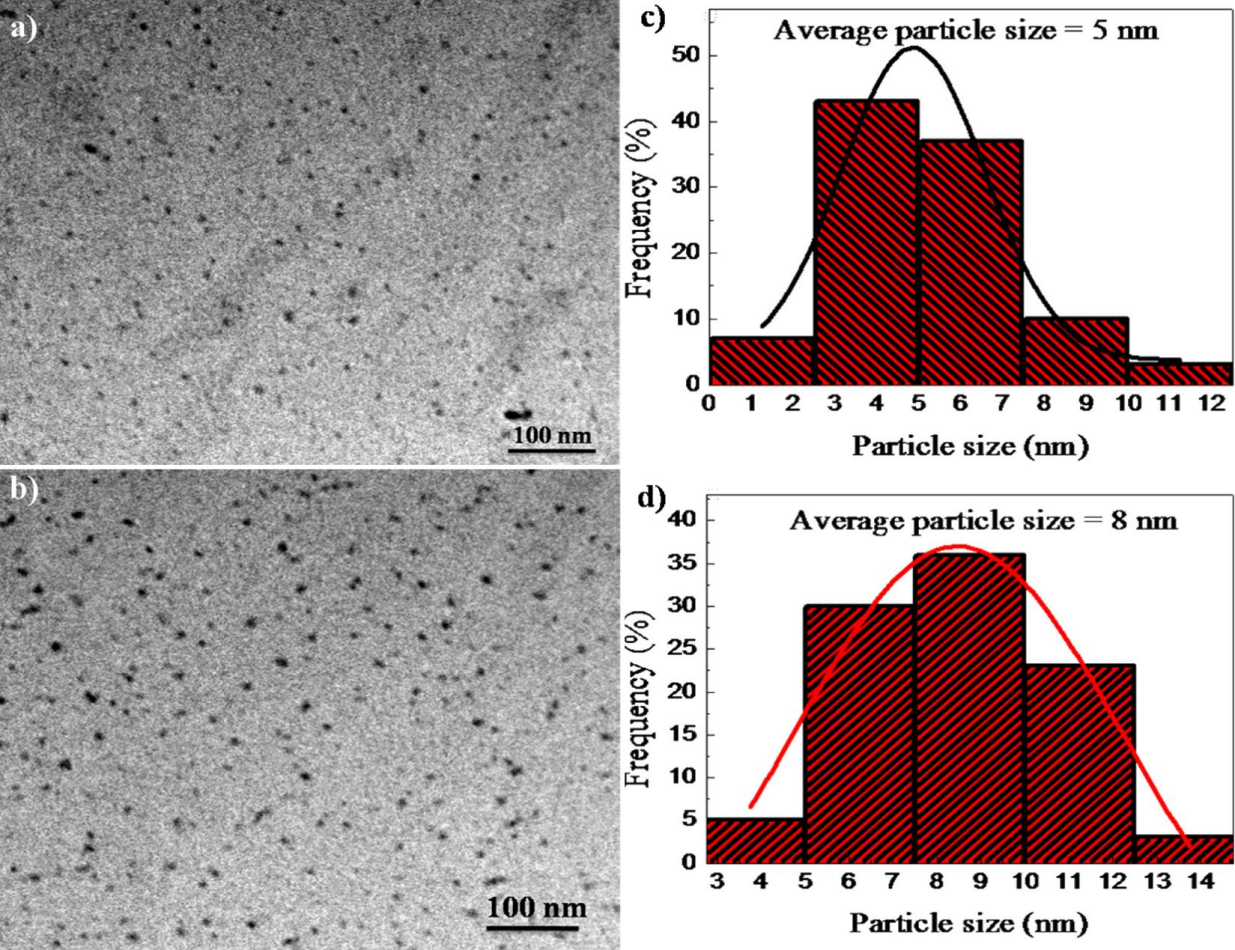
TEM micrographs of sputtered particles of (a) 2 atomic % Zn in silica and (b) 10 atomic % Zn in silica. (c) Size distribution of the particles corresponding to Figure 5a. (d) Size distribution corresponding to Figure 5b.

The sputtered species are collected on carbon-coated TEM grids placed on a catcher as shown in Figure 1. TEM pictures of the particles collected on the TEM grids for sample sets A and set B are shown in Figure 5a and Figure 5b, respectively. Particle size distributions corresponding to Figure 5a and Figure 5b are shown in Figure 5c and Figure 5d, respectively. The software Image J was used to calculate the size of the ejected particles [39]. The average sizes of the ejected particles are 5 nm for set A and 8 nm for set B, respectively. Thus, the average size of collected particles is nearly the same as that of the particles in the thin films after irradiation.

## Discussion

The main findings of the performed experiments are a large sputtering yield of Zn and its dependence on the size of the nanoparticles as well as the ejection of Zn clusters. The discussion of the above experimental results is given in the following sections.

### Large sputtering yield of Zn and its dependence on the size of nanoparticles

The large magnitude of sputtering yield can be explained on the basis of the inelastic thermal spike model. According to this model, a large amount of incident energy of swift heavy ions is transferred to the electrons of the target due to electronic excitations and ionizations. First, this energy is shared between the electrons of the target leading to the thermalization of the energy at a time scale of 10^−15^ to 10^−14^ s. Then the deposited energy is transferred from the electrons to the lattice through electron–phonon coupling. This coupling causes an increase in the lattice temperature of the target at a time scale of 10^−13^ to 10^−12^ s. The transient local temperature of the thermal spike depends on the volume, in which the energy is deposited and on the strength of the electron–phonon coupling. The electron phonon coupling factor (*g*) is given as [9]

[2]
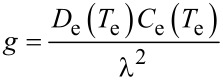


where *D*_e_ is the thermal diffusivity and *C*_e_ is the specific heat of the electronic system, λ is the mean free path of the excited electrons of the target. In the case of metals *g* is weak compared to insulators such as silica [40]. When a bulk metal is irradiated with SHI, the incoming ion transfers its energy to electrons of the target. Due to the high thermal conductivity, the deposited energy is quickly transferred to other electrons and the heat transferred to the metal lattice through electron–phonon coupling is not sufficient to cause the melting of the metal. Whereas in the case of silica, a smaller deposited energy is enough to cause melting due to the low thermal conductivity and the high value of *g*.

If we consider the case of metal nanoparticles embedded in silica (nanocomposite system), the scenario becomes quite different. The temperature of the metal nanoparticles embedded in silica raises above the melting point during irradiation [9,40] and leads to the following possibilities to cause sputtering.

**Figure 6:**
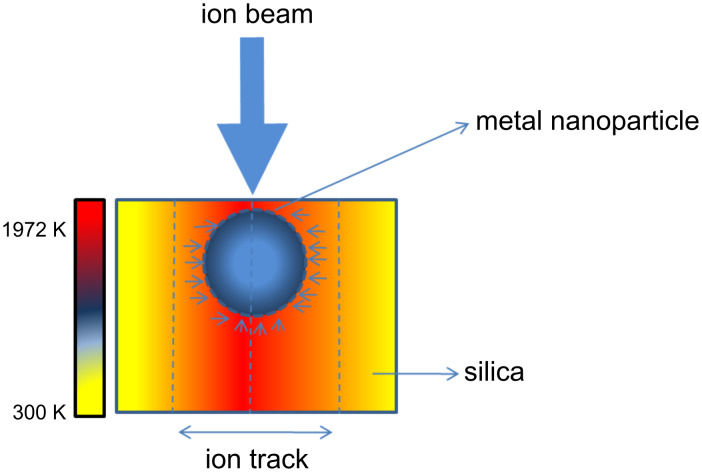
Schematic diagram of the formation of a thermal spike in the nanocomposite system. The small arrows around the metal nanoparticle correspond to the heat transferred to the nanoparticle from the surrounding molten silica.

**a:** When the transient temperature of the metal nanoparticle in silica is so high that the kinetic energy of the atoms is larger than the surface binding energy, then these atoms escape from the surface of the nanoparticle at the surface and near the surface region of the nanocomposite films and sputtering occurs. The temperature rise of metal nanoparticles in silica during SHI irradiation can occur in following ways. (i) The incident ions lose their energy through interaction with electrons of silica as well as of the metal nanoparticle. When the incident ion interacts with the electrons of the metal nanoparticle, the deposited energy rapidly diffuses to other electrons within the nanoparticle due to high thermal conductivity (ca. 318 W/(m·K) at 25 °C). This energy diffusion is then greatly hindered at the boundary of the metal nanoparticle because of the low thermal conductivity of surrounding silica. Therefore the deposited energy of electrons remains largely confined within the nanoparticle and causes the rise in overall lattice temperature of the nanoparticle. (ii) The interaction of the incident ions with the electrons of silica leads to a rise in its temperature above the melting point [40]. The surrounding molten silica with a temperature greater than 1972 K causes a temperature rise in the metal nanoparticle from its surface to the core as shown in Figure 6. (iii) Another way to look at the temperature rise in the metal nanoparticles embedded in silica provided by Awazu et al. [9]: The deposited energy is rapidly transferred among the electrons within the nanoparticle and then to the electrons of the surrounding silica, which causes a raise of the silica temperature. The electron–phonon coupling constant *g* of silica (about 10^13^ W/(cm^3^·K)) is larger than that of metals (about 10^10^ W/(cm^3^·K)), while its thermal conductivity is smaller [40]. Therefore the lattice temperature of silica increases more rapidly than that of the metal nanoparticle during irradiation. The heated silica feeds back the heat to the surface of the nanoparticle by phonon–phonon interaction, and the temperature of the metal nanoparticle raises above the melting point of 1342 K.

**b:** The other competing mechanism reported by several groups to cause high sputtering of metal nanoparticles embedded in silica is the ejection of clusters rather than the sputtering of atomic species. In the present case, TEM micrographs show that the sputtered species on the catcher are of the same size as the nanoparticles in the irradiated targets. This provides a direct evidence of cluster ejection. Ejection of direct clusters is explained on the basis of pressure spike generated inside the ion track. During SHI irradiation, the energy deposited by a fast ion leads to the creation of a cylindrical track along its path. The difference in pressure within the track and outside the track results in a pressure spike, which leads to the ejection of material from the surface of the solid material. A detailed discussion of the ejection of clusters is given in the next section.

The size-dependent sputtering yield can be explained on the basis of the energy density deposited per atom. For an incident ion traversing a spherical metal nanoparticle of radius *R*, it is given as [41]

[3]
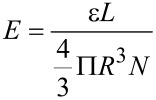


where ε is ion electronic energy loss in the nanoparticle, *L* is the ion path length in the nanoparticle and *N* is the atomic density. In the present case, nanoparticles are spherical in shape therefore ion path length in nanoparticle is 2*R*. Thus Equation 3 takes the form as

[4]
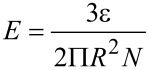


Therefore the temperature rise of the thermal spike is inversely proportional to the square of the size of the nanoparticle, considering that the temperature rise is proportional to *E*. Therefore, the lattice temperature of small nanoparticles raises much higher than that of large nanoparticles [9]. The higher lattice temperature of small nanoparticles results in increase of the kinetic energy of the atoms. Therefore, more atoms leave the surface and hence a higher sputtering yield per ion is observed in small nanoparticles as compared to large nanoparticles.

### Ejection of Zn clusters

The origin of sputtered species on catcher of the same size as those in irradiated target samples can be explained in two ways. The sputtered species collected on the catcher can be in atomic form and these atoms coalesce to form clusters due to surface diffusion on the catcher. The other possible way is that during irradiation the clusters are directly ejected from the target surface [24]. A shock wave model [18] was proposed to theoretically explain the emission of clusters during irradiation. According to this model overlapping collision cascades in the low energy regime can result in shock waves which propagate to the material surface and result in the emission of chunks of material from the surface. Kuiri et al. [25] described the ejection of clusters in the high energy regime on the basis of an universal aggregation mechanism that shows a power-law decay with δ values of 3/2 for small clusters and 7/2 for larger clusters, respectively. This rules out any possibility of a liquid–gas phase transition taking place in the vaporized Au nanoparticle resulting from SHI irradiation. Johnson et al. [42] reported a unifying description of sputtering in three different regimes of energy and in each regime in ascending order of energy, different ejection mechanism takes place, i.e., single events, diffusive spike and pressure pulse. There are several other mechanisms suggested to describe how the energy deposited by the incident ion to the target atoms can result in a cluster ejection [42–44]. It has been proposed in the present case that when an ion strikes the target it creates a cylindrical track along its path. According to thermal spike model the ion track in silica is created for picoseconds and in this period the temperature of silica raises above its melting point. It is well known that a material in molten state has lower density than in the solid state. Due to the difference in density of molten and solid material it gives rise to a pressurized cylindrical disturbance, referred to as pressure pulse or pressure spike along the track. To release this pressure, the cylinder undergoes a volume expansion but the expansion is not possible in a radial direction and hence the expansion occurs vertically upward into the vacuum [45]. At the vacuum target interface, the volume expansion results in the ejection of material from the surface. In the present case, the solid material consists of Zn clusters embedded into silica, which eject due to the pressure spike generated inside the track. Therefore we observed clusters collected on the catcher that are similar in size to those in the target. There are evidences of pressure spikes in the jet-like emission of sputtered species in LiF [12].

## Conclusion

Zn–silica nanocomposite thin films with two different metal contents were irradiated with 100 MeV Ag ions. Sputtering during irradiation is found to have an inverse dependence on the nanoparticle size. It is explained on the basis of a thermal spike model and it is proposed that the lattice temperature of small nanoparticles increase much more that of larger nanoparticles. Sputtered particles at the catcher have the same size as that in the nanocomposite film, indicating that clusters eject during irradiation. Ejection of clusters during irradiation is due to the pressure spike inside the ion track, initiated by a thermal spike.
